# Supplementary material for: Relationship between Baseline Serum Potassium and 1-Year Readmission in Pediatric Patients with Heart Failure: A Retrospective Cohort Study
Source: Children (Basel). 2024 Jun 14;11(6):725. doi: 10.3390/children11060725 (PMC11201687; doi:10.3390/children11060725)
Supplement: Supplementary file 1 [file children-11-00725-s001.zip › children-2977703-supplementary.pdf]

Supplementary Figure S1

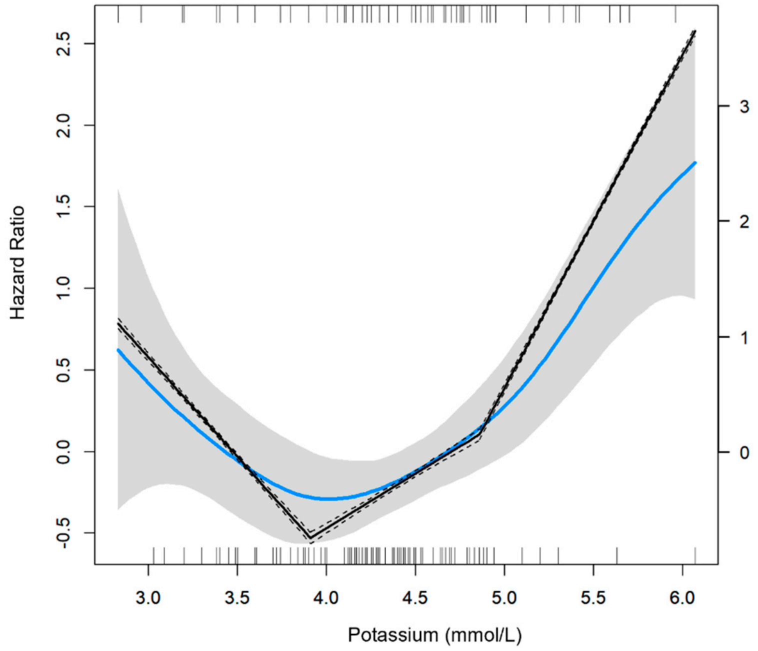

Figure S1: A Cox inflection point analysis of potassium levels and 1-year readmission.

Note: No variables were adjusted.

Supplementary Table S1 The collinearity diagnostic analysis of 1-year readmission in pediatric patients with heart failure

Variables	Tolerance	VIF
Age	0.430	2.325
Sex	0.947	1.056
SCr	0.420	2.383
BUN	0.646	1.548
SUA	0.657	1.523
eGFR	0.899	1.112
Serum potassium	0.889	1.125
Serum sodium	0.780	1.283
ALB	0.905	1.105
LVEF	0.937	1.067
NYHA/Ross classification	0.793	1.261

Abbreviations: SCr serum creatinine, BUN blood urea nitrogen, SUA serum uric acid, eGFR estimated glomerular filtration rate, ALB serum albumin, LVEF left ventricular ejection fractions; NYHA New York Heart Association.
